# Patient-derived zebrafish xenografts of uveal melanoma reveal ferroptosis as a drug target

**DOI:** 10.1038/s41420-023-01446-6

**Published:** 2023-06-16

**Authors:** Arwin Groenewoud, Jie Yin, Maria Chiara Gelmi, Samar Alsafadi, Fariba Nemati, Didier Decaudin, Sergio Roman-Roman, Helen Kalirai, Sarah E. Coupland, Aart G. Jochemsen, Martine J. Jager, Felix B. Engel, B. E. Snaar-Jagalska

**Affiliations:** 1 Institute of Biology, Leiden University, Leiden, The Netherlands; 2 Experimental Renal and Cardiovascular Research, Department of Nephropathology, Institute of Pathology, Friedrich-Alexander-Universität Erlangen-Nürnberg (FAU), Erlangen, Germany; 3 Bavarian Cancer Research Center (BZKF), 91054 Erlangen, Germany; 4 Comprehensive Cancer Center Erlangen-EMN (CCC ER-EMN), Erlangen, Germany; 5 Department of Ophthalmology, Leiden University Medical Center, Leiden, The Netherlands; 6 Uveal Melanoma Translational Group, Department of Translational Research, Institut Curie, PSL Research University, 75248 Paris, France; 7 Laboratory of Preclinical Investigation, Translational Research Department, Institut Curie, PSL Research University, 75248 Paris, France; 8 Liverpool Ocular Oncology Research Centre, Department of Molecular and Clinical Cancer Medicine, University of Liverpool, Liverpool, United Kingdom; 9 Department of Cell and Chemical Biology, Leiden University Medical Center, Leiden, The Netherlands

**Keywords:** Cancer models, Cell death

## Abstract

Uveal melanoma (UM) has a high risk to progress to metastatic disease with a median survival of 3.9 months after metastases detection, as metastatic UM responds poorly to conventional and targeted chemotherapy and is largely refractory to immunotherapy. Here, we present a patient-derived zebrafish UM xenograft model mimicking metastatic UM. Cells isolated from Xmm66 spheroids derived from metastatic UM patient material were injected into 2 days-old zebrafish larvae resulting in micro-metastases in the liver and caudal hematopoietic tissue. Metastasis formation could be reduced by navitoclax and more efficiently by the combinations navitoclax/everolimus and flavopiridol/quisinostat. We obtained spheroid cultures from 14 metastatic and 10 primary UM tissues, which were used for xenografts with a success rate of 100%. Importantly, the ferroptosis-related genes *GPX4* and *SLC7A11* are negatively correlated with the survival of UM patients (TCGA: *n* = 80; Leiden University Medical Centre cohort: *n* = 64), ferroptosis susceptibility is correlated with loss of BAP1, one of the key prognosticators for metastatic UM, and ferroptosis induction greatly reduced metastasis formation in the UM xenograft model. Collectively, we have established a patient-derived animal model for metastatic UM and identified ferroptosis induction as a possible therapeutic strategy for the treatment of UM patients.

## Introduction

Uveal melanoma (UM) is an aggressive and deadly ocular cancer, derived from melanocytic cells of the uvea (made up of the iris, choroid, and ciliary body) [1, 2]. Between 7–33% of all primary UM patients develop deadly metastatic disease within 10 years, which is strongly linked to mutations in the BRCA-associated-protein 1 (BAP1) gene [2, 3]. Importantly, the prognosis of metastatic UM patients is grim, with a median survival of 3.9 months after detection of metastases [4], as metastatic UM responds poorly to conventional and targeted chemotherapy and in contrast to cutaneous melanomas, is largely refractory to immunotherapy [5–7]. From a genetic point of view, UMs almost obligately carry an activating mutation in G protein-coupled receptor (GPCR) genes encoding proteins of the G protein-coupled subunit alpha (GNA) family (mainly in *GNAQ* and *GNA11*) blocking GTPase activity, effectively driving oncogenic hyperactivation of Gq or G_11_1. This hyperactivation leads to a subsequent increase in downstream signaling, including the protein kinase C (PKC)/MAP kinase/ERK signaling axis [8, 9]. In addition, UM is characterized by strong prognosticators such as monosomy 3 [10–14] and the loss of expression of the BAP1 gene located on chromosome 3, which is usually accompanied by the loss of chromosome 3 [3].

Despite the lethal nature of metastatic UM, there is currently no animal model available to study metastatic UM and/or to identify potential drugs or drug targets for therapy. Past research is based mainly on 2D cell culture system of subcutaneous xenografts in the mouse, which do not result in metastatic spread [15, 16]. In recent years, the zebrafish xenograft model has been established as valuable model for mechanistical studies and pre-clinical drug assessment in the cancer field [17–19]. The basic idea of the zebrafish xenograft model is to inject human cancer cells into the vasculature of larvae, like in most murine models, from where they disseminate passively through blood flow. Similar to humans and murine models, the cells anchor to an endothelial bed, both passively due to physical entrapment and actively by deploying cell-cell adhesions, facilitating perivascular-metastasis initiation and subsequent extravasation. Zebrafish larvae have several advantages when compared to “conventional” cancer model organisms such as: i) high level of optical transparency; ii) no host-graft rejection due to the lack of a fully developed adaptive immune system; iii) metastasis formation within a few days; iv) availability of a large number of fluorescent reporter zebrafish lines; v) up to several hundred progeny per mating pair per week; vi) *ex utero* fertilization; vii) high homology to humans (70% of the genes), whereby 85% of all human disease-related genes are conserved [20]; iix) amenable for high throughput screening and genetic manipulation. Collectively, the zebrafish model does not only allow to efficiently visualize the entire process of early metastasis through live high-resolution imaging, on a whole animal level, but also to modulate the process to elucidate the mechanisms controlling the process of early metastasis in order to identify and validate potential drug targets.

A simple and predictive animal model for metastatic UM is important to better understand the underlying mechanisms and to test hypothesis-driven potential therapeutic drugs. For example, over 90% of UM carry somatic *GNAQ/11* mutations which are known to activate the RAS-MAP kinase pathway [8]. On the other hand it is known that cells that are exposed to oxidative stress [21] and exhibit oncogenic hyperactivation of the RAS-signaling cascade are sensitized to ferroptotic cell death due to an intrinsic de-regulation of iron homeostatic mechanisms [22]. Recent discoveries have uncovered the role of ferroptosis in the suppression of metastasis development [21, 23], contributing to the elimination of 90–99% of all circulating cancer cells before they find a suitable metastatic niche [24–26]. Ferroptosis is a non-apoptotic form of regulated cell death that is caused by iron-mediated overproduction of lipid-based reactive oxygen species (ROS), particularly lipid hydroperoxide [27]. Glutathione peroxidase 4 (GPX4) reverts lipid peroxides back to their unoxidized form inhibiting ferroptosis which depends on its substrate glutathione [28]. Notably, SLC7A11, the catalytic subunit of the cystine/glutamate antiporter (system Xc−), is the major transporter of extracellular cystine, which intracellularly is rapidly converted to cysteine serving as the precursor for glutathione synthesis. Thus, cancer cells that express high levels of GPX4 and/or SLC7A11 are protected from ferroptosis. Notably, it has been reported that the expression of SLC7A11 is inhibited by BAP1 [29] providing an explanation why loss of BAP1 is a key prognosticator for metastatic UM and suggesting that induction of ferroptosis in metastatic UM is a promising treatment option.

Here we report the generation of patient-derived UM spheroids which allow UM metastasis formation in zebrafish upon engraftment of spheroid cells into the circulation of zebrafish larvae. Utilizing this model, we confirmed our hypothesis - based on the negative correlation of the expression of the ferroptosis-related genes GPX4 and SLC7A11 with survival of UM patients - that conventional ferroptosis activators are potent inducers of ferroptotic UM cell death in vivo.

## Results

### Spheroid cultures of UM patient tissues retain metastatic features

**Fig. 1:**
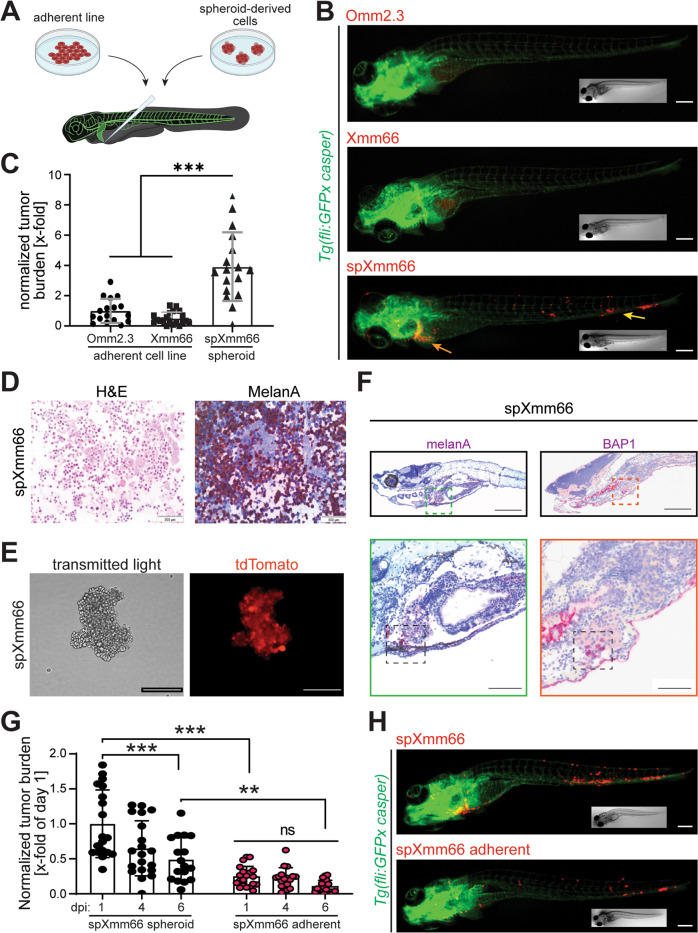
Generation of highly metastatic uveal melanoma in zebrafish from spheroid cultures. **A** Scheme representing the approach to inject metastatic uveal melanoma in zebrafish. Spheroid cultures are reduced to single cell suspensions by enzymatic dissociation, and single cells are injected through the Duct of Cuvier (doC) into the blood circulation of 48 hpf *Tg(fli:GFPx casper)* zebrafish larvae, in which all vessels are labelled with GFP (green). **B** Stereomicroscopic images of representative phenotypes of *Tg(fliGFPx casper)* zebrafish larvae 6 days post injection with lentivirally labelled cells (red) derived from the commonly used UM cell lines Omm2.3 and Xmm66 compared to spheroid-derived Xmm66 cells (spXmm66). Scale bars: 100 μm. Inserts: same fish imaged with bright field microscopy. **C** Quantitative analysis of the metastatic capacity of adherent uveal melanoma Omm2.3 and Xmm66 cells and UM-derived spheroid line spXmm66 upon engraftment into zebrafish (n = 20). Data are mean ± SD. **D** Analysis of spXmm66 by H&E as well as melanA staining (marker for the melanocytic origin of the engrafted cells). **E** Microscopic images of the spXmm66 spheroid line in suspension after lentiviral transduction resulting in tdTomato expression. **F** Representative images of tissue sections of zebrafish engrafted with spXmm66 cells 6 days post injection stained for hematoxylin and eosin and BAP1 (dark purple, boxed area) or melanA (dark purple, boxed area). Scale bars: 1 mm (overview image), 500 µm (magnification). **G** Stereomicroscopic images of representative phenotypes of *Tg(fliGFPx casper)* zebrafish larvae 6 days post injection with tdTomato-labelled spXmm66 cells grown in suspension (spheroid) or as de novo adherent cultures (7 days conventional cell culture on plastic). Scale bars: 100 μm. Inserts: same fish imaged with bright field microscopy. **H** Quantitative analysis of the metastatic capacity of spXmm66 cells grown in suspension (spheroid) or as de novo adherent cultures upon engraftment into zebrafish (*n* = 20). Data are mean ± SD. ns: not significant. **:*p* < 0.01; ***:*p* < 0.001.

Currently, no patient-derived metastatic UM animal model is available. Therefore, we tested whether intravenous injection of cells of the adherent growing patient-derived metastatic UM cell line Xmm66 [30] into zebrafish results in the formation of metastases. For this purpose, the cells were lentivirally labeled (red) and injected via the Duct of Cuvier into 48 hours post fertilization (hpf) *Tg(fli:GFP x casper)* embryos, in which all vessels are labeled in green (Fig. 1A). Immunofluorescence analysis and quantification of total tumor burden at 6 days post injection (dpi) revealed a low engraftment rate (Fig. 1B, C). Tumor burden is a measure of normalized total fluorescence, normalized to either fluorescence values of 1dpi or to an internal control. Similar results were obtained with Omm2.3 cells, another adherent UM cell line [31]. Thus, this system does not represent a robust system for drug screening or elucidating molecular mechanisms underlying UM metastasis formation.

In the past, it has become clear that cells cultured in 3D maintain their in vivo tumorigenic potential to a greater extent [32]. Importantly, it has been shown for several different cancer types (e.g., liver cancer [33], colon cancer [34], osteosarcoma [35], leukemia [36]) that the properties of cancer stem cells, which are a rare tumor subpopulation with high differentiation, proliferative, tumorigenic, and metastatic potential compared to other tumor cell populations, are maintained in 3D culture (especially as spheroids) [37, 38]. Therefore, we tested whether cells generated from Xmm66 spheroids (Fig. 1D, E), spXmm66 cells, exhibit an enhanced tumorigenic/metastatic potential compared to adherent Omm2.3 and Xmm66 cells. For this purpose, we established one stable spheroid line (spXmm66) from the same donor of which the adherent cell line Xmm66 was generated from [30]. spXmm66 cells maintained melanA expression, indicating the melanocytic origin of the engrafted cells (Fig. 1D) and spheroids could efficiently be lentivirally labelled (Fig. 1E). Analysis of the tumor burden at 6 dpi revealed that injection of spXmm66-derived cells resulted in a significant higher tumor burden with metastasis formation in the region of the liver (Fig. 1B, yellow arrow) and the caudal hematopoietic tissue of the zebrafish (analogous with the mammalian fetal liver [39, 40], Fig. 1B, orange arrow). Thus, spXmm66 cells appear to spread to liver tissue, as usually seen in the majority of patients (~90%) [41]. To validate metastasis formation in the liver, paraffin-embedded zebrafish were sectioned and stained for the melanocyte-specific marker melanA and for the presence of BAP1, one of the key prognosticators for metastatic UM. All engrafted larvae showed melanA and BAP1 staining in the liver (Fig. 1F). Notably, if spXmm66 cells were cultured adherent as mono-layer, their tumorigenic/metastatic potential was significantly reduced and no obvious metastases were observed in the liver (Fig. 1G, H).

**Fig. 2:**
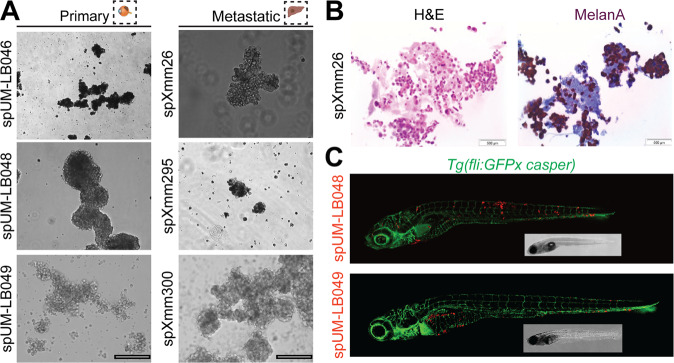
Spheroid cultures can readily be established from both primary uveal melanoma tumors and patient-derived metastatic uveal melanoma tissues derived from murine xenografts. **A** Representative image of the established spheroid cultures. Primary: uveal melanoma patient tumor tissue. Metastatic: murine xenograft material, derived from metastatic UM samples propagated subcutaneously. Scale bars: 250 µm). **B** Analysis of spXmm26 by H&E as well as melanA staining (marker for the melanocytic origin of the engrafted cells). **C** Stereomicroscopic images of representative phenotypes of *Tg(fliGFPx casper)* zebrafish larvae (blood vessels: green) 6 days post injection with CM-DiL-labelled spheroid cells (spUM-LB048 and spUM-LB049, red) derived from primary uveal melanoma tumor tissues. Note, disseminated cancer cells are present up to 6 days post engraftment and settle in both the hematopoietic tissue and/or the liver. Inserts: same fish imaged with bright field microscopy.

To assess whether spheroids can be efficiently generated from patient-derived UM material, spheroids were generated from primary tumor-derived tissue from 10 patients and metastatic tumor-derived tissue from 14 patients (Supplementary Table 1). All primary tumor-derived tissue samples readily formed spheroids in culture (100% success rate) within 24 hours and were cultured for 3–7 days (Fig. 2A), maintaining melanA expression (Fig. 2B). To determine whether spheroid-derived cells in general allow metastasis formation upon injection into zebrafish larvae, cells from primary tumor-derived spheroids from 2 additional patients were injected into zebrafish embryos. In both cases cell injections resulted in metastasis formation, similar to spXmm66 cell injections (Fig. 2C). Out of the 14 metastatic tumor-derived tissues, 13 spheroid cultures were successfully maintained as short-lived spheroid cultures (at least 7 days, 100% success, Supplementary Table 1), in addition to the stable spheroid line spXmm66 (Fig. 1). Notably, all tested primary tissues (between 2.5–5 mm^3^ sample size during enucleation) yielded enough material after short-lived spheroid culture for at least two larval zebrafish engraftments on different days, using different genetically distinct clutches (at least 80 injected individuals per experiment) within 7–14 days after establishment.

Taken together, we have successfully established a platform to isolate, preserve, and recover viable spheroid cultures that allow to model metastatic spread of human UM cells in the zebrafish into the liver.

### The human metastatic UM zebrafish model is a suitable drug screening tool

**Fig. 3:**
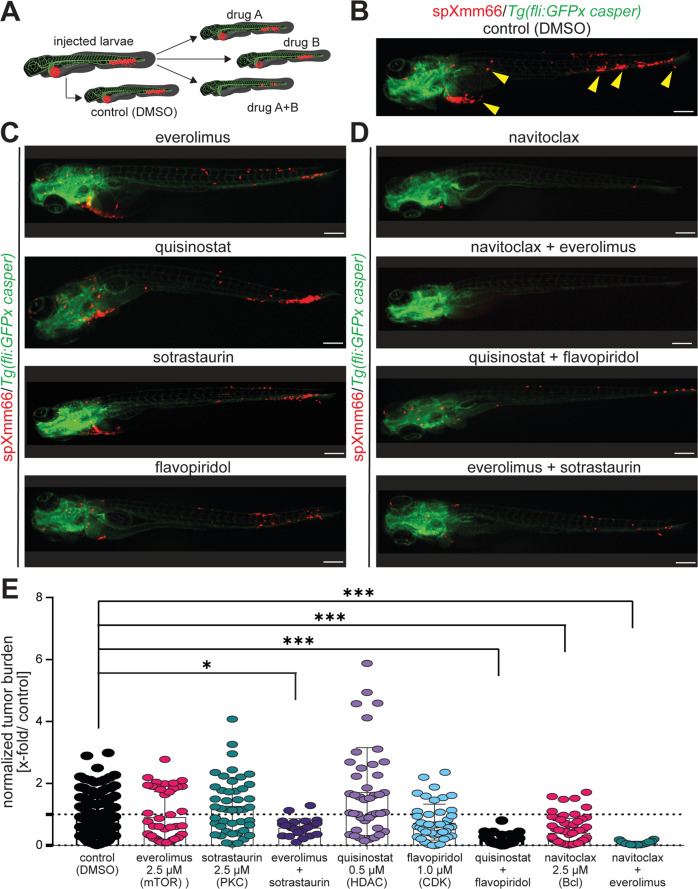
Metastatic uveal melanoma zebrafish model is suitable for drug screening. **A** Scheme representing the approach to perform drug screening in the here established metastatic uveal melanoma zebrafish model utilizing spXmm66 cell engraftment at 48 hpf followed by tumor burden analysis 6 dpi. **B**–**D** Stereomicroscopic images of representative phenotypes of *Tg(fliGFPx casper)* zebrafish larvae 6 days post injection with tdTomato-labelled spXmm66 cells and treatment with the indicated drugs at their maximum tolerated dose (>80% survival, Supplementary Fig. 1). Scale bars: 250 µm. **E** Quantitative analysis of (**B**–**D**) to determine the effect of the indicated drug treatment on tumor burden, normalized to DMSO control (normalized tumor burden). Data are mean ± SD. *:*p* < 0.05; **:*p* < 0.01; ***:*p* < 0.001.

The zebrafish xenograft model has been established as valuable model for mechanistical studies and pre-clinical drug assessment [17–19]. To evaluate the suitability of our metastatic UM zebrafish model based on spXmm66 cells, the effect of several small molecule inhibitors was tested on metastasis formation. These inhibitors reduced tumor progression alone or in combination in female SCID mice xenografted with a tumor fragment of 20–40 mm^3^ derived from liver metastases (*n* = 5) or cutaneous metastasis (*n* = 1) [42] and/or reduced in vitro growth of several UM cell lines, including Xmm66 cells [43, 44]. For this purpose, the maximum tolerated dose of each selected drug was determined as previously described (≥80% survival) [45] by treating non-injected zebrafish larvae at 72 hpf for 5 days (Supplementary Fig. 1). Drug treatment was renewed every other day by changing the drug-laced zebrafish medium. Subsequently, spXmm66-engrafted zebrafish were treated 16 hours post injection with the drugs and concentrations indicated in Fig. 3, individually and in combination. After 5 days, the effect of the drug treatments on tumor burden was determined. Compared to the treatment with the diluent DMSO (Fig. 3B) and treatments indicated in Fig. 3C, navitoclax (BCL-2/BCL-xl inhibitor) as well as the combinations navitoclax + everolimus (mTORC1 inhibitor) and quisinostat (HDAC inhibitor) + flavopiridol (CDK inhibitor) resulted in a significant and marked reduction in tumor burden, while everolimus + sotrastaurin (PKC inhibitor) resulted in a significant but moderate reduction (Fig. 3D, E). Collectively, we conclude that the here presented model system is suitable as drug screening tool whereby in contrast to the subcutaneous mouse model utilizing adherent cells not all drugs and drug combinations were successful. In concordance with these findings, our in vitro ferroptosis induction data (Supplementary Fig. 4), indicate that conventional ferroptosis induction does not robustly result in a strong reduction in cell proliferation. Moreover, the exceedingly high concentrations of both RSL3 and erastin required for significant growth reduction in vitro (>4 µM) imply that an external factor might be required (such as Fe^2+^).

### Ferroptosis detoxification gene expression correlates inversely with UM patient survival

**Fig. 4:**
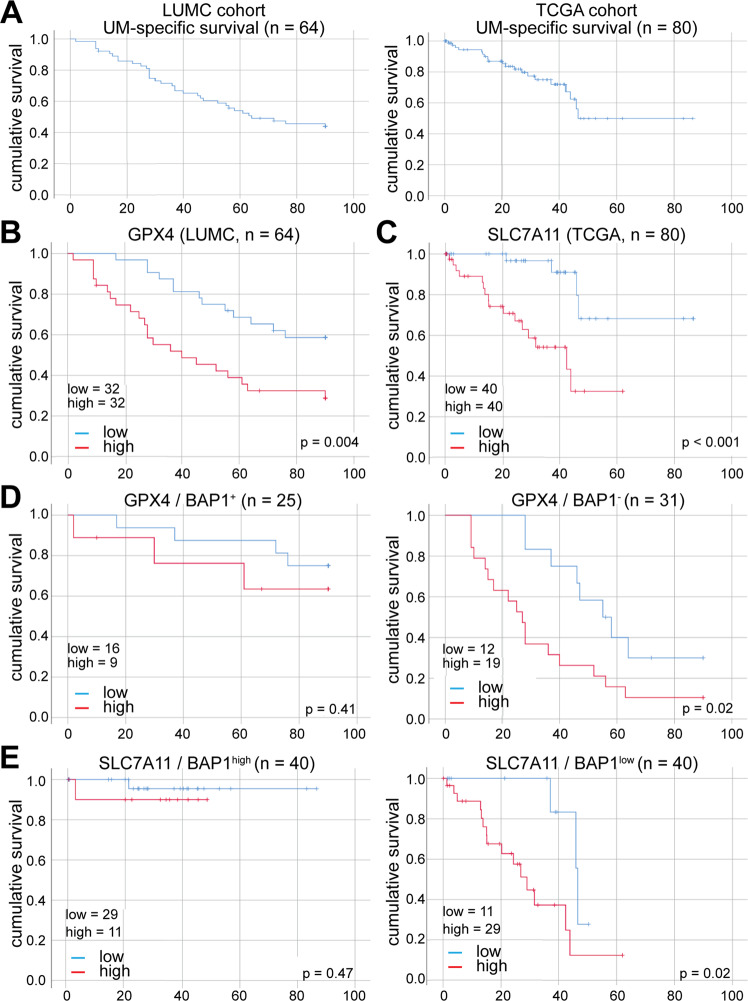
Ferroptosis-related genes negatively correlate with uveal melanoma patient survival. **A**, Analysis of the UM-specific survival in both LUMC and TCGA patient cohorts. **B**, **C** High GPX4 (**B**) and SLC7A11 (**C**) expression levels (divided over the median) show a correlation with reduced patient survival (*p* = 0.004 and *p* = 0.0014, respectively). **D**, **E** Comparative analysis of the relation between GPX4 (**D**) and SLC7A11 (**E**) and survival in BAP1^+^ (LUMC, determined by IHC, *n* = 25) and BAP1 high (TCGA, determined by RNAseq, *n* = 40) UM samples compared to survival in BAP1^-^ (LUMC, IHC, *n* = 31) and BAP1 low (TCGA, RNAseq, *n* = 40) UM samples. The expression levels of GPX4, SLC7A11 and BAP1 were split at the median, and curves were plotted using SPSS.

Recently, it has been shown that for several cancer types, including cutaneous melanoma [21], that oxidative stress inhibits metastasis formation through induction of ferroptosis, eliminating circulating cancer cells in the circulation. Therefore, we wondered whether an upregulation of ferroptosis detoxifying mechanisms might play a role in UM disease progression. To test this hypothesis, we utilized two cohorts of UM, the Leiden University Medical Centre (LUMC) cohort (*n* = 64) and the TCGA cohort (*n* = 80) (Fig. 4A). We analyzed the relation between expression of the three major eukaryotic ROS detoxifying enzymes catalase (CAT), superoxide dismutase 2 (with the expression of the glutamate/cysteine antiporter SLC7A11, which plays an important role in intracellular iron metabolism known to affect ferroptosis. While no correlation was found for CAT and SOD2 in the TCGA cohort (Supplementary Fig. 2, note no data are available for LUMC cohort), the analysis revealed that GPX4 expression negatively correlates with UM-specific survival in the LUMC cohort (*p* = 0.004) (Fig. 4B) and a strong negative correlation with patient survival was identified for SLC7A11 in the TCGA cohort (*p* < 0.001) (Fig. 4C). These data suggest that ferroptosis plays an important role in metastasis formation in UM.

To substantiate our hypothesis that ferroptosis plays an important role in metastatic UM, we assessed whether GPX4 and SLC7A11 expression negatively correlates with survival of UM patients with low expression of BAP1 (BAP^−^). In contrast to BAP1^+^ patient survival, GPX4 and SLC7A11 expression showed an enhanced negative correlation with survival in BAP1^−^ patients (GPX4: *p* = 0.02 vs. *p* = 0.41; SLC7A11: *p* < 0.02 vs. *p* = 0.47, Fig. 4D, E).

**Fig. 5:**
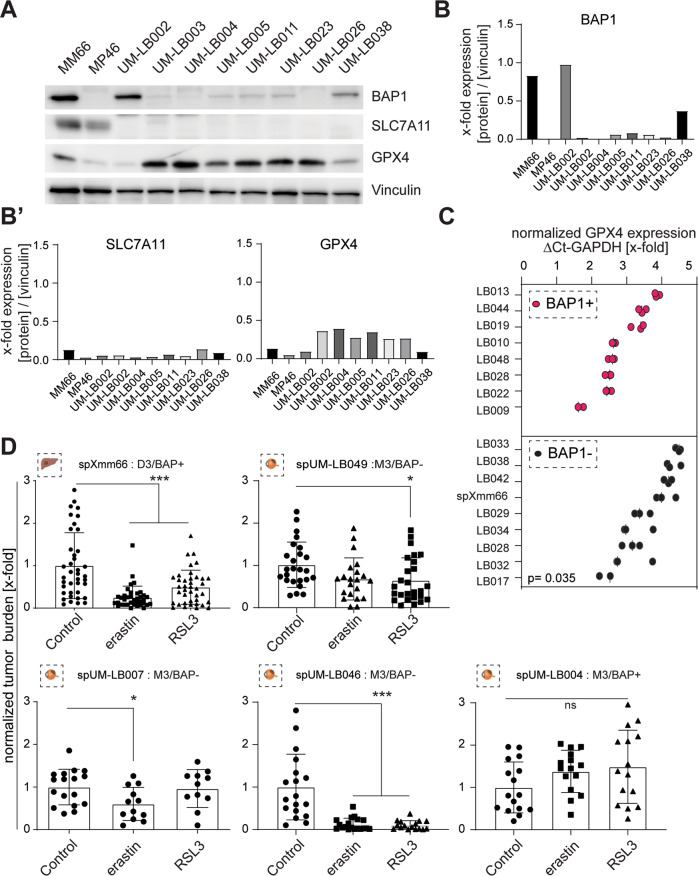
Ferroptosis induction inhibits metastasis formation in the metastatic uveal melanoma zebrafish model. **A** Western blot analysis determining GPX4, SLC7A11 and BAP1 protein expression in the two established cell lines MM66 and MM 46 and eight patient-derived tissues. Loading control: vinculin. Note, while BAP1 and GPX4 show a negative correlation in the patient samples, SLC7A11 was barely detected, independent of BAP1 expression levels. **B**, **B**’ Westernblot quantification of BAP1, SLC7A11 and GPX4. **C** qPCR analysis of *GPX4* mRNA expression in primary UM tissues and spXmm66 (green dots), with known BAP1 status. Expression values were normalized to GAPDH (ΔCT). *n* = 3. **D** Quantitative analysis of tumor burden in the here established metastatic uveal melanoma zebrafish model. The indicated cells were engrafted at 48 hpf, maintained for 6 days in the absence or presence of the ferroptosis inducer erastin (SLC7A11 inhibitor, 5 μM) or RSL3 (GPX4 inhibitor, 10 μM) and subsequently subjected to tumor burden analysis. Notably, ferroptosis induction via erastin and/or RSL3 resulted in 4 out of 5 cases in a significant reduction of tumor burden. *n* = 40 (spXmm66) or *n* = 20 (all other cases). Data are mean ± SD. D3: chromosome 3 disomy: M3: chromosome 3 monosomy. ns: not significant. ***:*p* < 0.001.

To validate a negative correlation between BAP1 expression and the ferroptosis-related proteins GPX4 and SLC7A11, we examined a panel of 8 primary UM patient samples. These samples were compared to the established metastatic UM cell lines MM66 (BAP1 positive) and MP46 (BAP1 negative) (Fig. 5A). We found that both cell lines, MM66 and MM46, showed highly-elevated protein levels of SLC7A11 compared to the patient-derived samples (BAP1-positive and -negative samples) in which no SLC7A11 was detected. Thus, no correlation was found between BAP1 and SLC7A11 expression. In contrast, the primary patient samples showed an inverse correlation between BAP1 and GPX4 expression levels. The samples UM-LB002 and UM-LB038 contained the highest BAP1 and by far lowest GPX4 expression levels. These results were confirmed in parallel experiments in which we performed a confirmatory qPCR-based analysis of GPX4 expression for two primary UM patient cohorts (BAP1^+^ = 8, BAP1^−^ = 8 + spXmm66). These results revealed that GPX4 high and low expression populations could be segregated based on their BAP1 status (*p* = 0.035, Fig. 5B).

In summary, our analysis reveals a strong inverse correlation between a key prognosticator for metastatic UM, BAP1, and the ferroptosis-related gene *GPX4* indicating that ferroptosis plays an important role in metastatic UM progression.

### Ferroptosis induction reduces UM metastasis

To determine whether ferroptosis induction can inhibit metastatic outgrowth of UM cells, we first established the maximum tolerated dose for the ferroptosis inducer erastin (SCL7A11 inhibitor, 5 µM) and RSL3 (GPX4 inhibitor, 10 µM) (Supplementary Fig. 1C). Subsequently, 36 larvae were engrafted with cells from spheroids derived from one metastatic (spXmm66) and four primary UM tissues and then treated with erastin and RSL3 to induce ferroptosis by inhibiting SCL7A11 and GPX4, respectively. Per condition, ±20 zebrafish larvae were imaged after 6 days of treatment to determine tumor burden (Fig. 5). This analysis revealed that RSL3 treatment inhibited significantly metastasis formation (tumor burden) upon engraftment of spXmm66 cells as well as cells from spheroids derived from two primary UM tissues (spUM-LB046, spUM-LB049). Notably, these primary UM tissues were scored in the clinic BAP1^−^/monosomy 3 and thus should express increased levels of GPX4 (note that tumor sample sizes did not allow in all cases to perform engraftment experiments plus expression analysis) which explains the sensitivity of the cells to RSL3 induced ferroptosis. Importantly, although spXmm66 is derived from a BAP1^+^/non-monosomy 3 (D3) tumor it highly expresses GPX4 (Fig. 5B), explaining its strong response to RSL3. In contrast, tumor burden was not decreased by RSL3 treatment in zebrafishes engrafted with spUM-LB004 which was scored in the clinic BAP1^+^. However, our western blot analysis showed at best low BAP1 expression levels but an extremely high GPX4 expression level. Thus, the chosen RSL3 concentration might not have been high enough to induce ferroptosis.

Similar to RSL3, erastin inhibited tumor burden in zebrafish engrafted with spXmm66, spUM-LB046 and spUM-LB007 cells (Fig. 5). The lower efficacy of erastin might be due to the fact that in the analyzed 8 tumor samples SCL7A11 expression levels were very low. Yet, that erastin can inhibit metastasis formation might be due its inhibitory effect on VDAC in mitochondria resulting to increased levels of ROS which also promotes ferroptosis [23].

In conclusion, using this zebrafish model, we have been able to demonstrate that both metastatic and primary UM cells were susceptible to pharmacological induction of ferroptosis. Moreover, we have shown a possible predictor for ferroptosis treatment response in both clinically relevant and routinely detected UM markers BAP1 and monosomy 3, indicating that these patients could benefit from pro-ferroptotic therapy. Finally, GPX4 appears to be a new strong predictor for pro-ferroptotic therapy efficacy.

## Discussion

We conclude that the here developed zebrafish model of human metastatic UM is a suitable model for the identification of potential drug targets of metastatic UM and that clinical treatment with ferroptosis inducers after BAP1 stratification of UM patients is a promising strategy.

While the zebrafish is an excellent system to study the behavior of adherent growing human metastatic cancer cells in vivo, metastatic UM cells failed to colonize the zebrafish post injection. The underlying reason is unclear. The argument that 3D-culturing maintains stemness of cancer stem cells would be valid for all cancer types. A possible explanation might be that UM tissues contain a relative low number of cancer stem cells. Another explanation might be that the maintenance of UM metastatic capacity is dependent on extrinsic environmental factors, that are lost after explantation and in vitro cultivation. In future studies, it will be important to determine differences in differentiation and cell signaling between 2D- and 3D- cultured UM cells but also to assess whether the 3D culture of other patient-derived tumor cells exhibit an increased metastatic potential compared to in parallel established adherent cells.

Notably, while we were able to generate from all patient-derived material (*n* = 24) spheroid cultures, almost all of them exhibited low proliferation rates and thus were short-lived and could not be maintained. The reason for these differences remains unclear. However, all spheroid cells engrafted in zebrafish (in total from 5 different patients) resulted in metastasis formation.

Here, we established an in vivo model to study metastatic UM. This is important as our data demonstrate that adherent growing cells are not metastatic upon engraftment into zebrafish and thus are not a reliable system to predict drug efficacy in the context of UM. In addition, drug response depends often on environmental factors which are either not considered or are difficult to simulate in vitro. For example, ferroptosis depends on the presence of factors such as ferrous iron (Fe_2_^+^) as well as an oxidative and mechanically challenging environment. The fact that not all drugs and drug combinations that reduced tumor progression in a subcutaneous female SCID mice xenograft model [30, 42, 43] or reduced in vitro growth of several UM cell lines [42] further underlines the importance of our zebrafish xenograft model mimicking metastatic UM.

Our analysis of TCGA and LUMC data indicates the presence of a strong inverse correlation of the ferroptosis-related genes GPX4 and system Xc- (SLC7A11) with (metastasis-free) patient survival. This is in agreement with the recent identification of a robust ferroptosis-related seven-gene signature for UM which is strongly associated with the prognosis of UM and can precisely detect UM risk level.

Importantly, we have here shown that induction of ferroptosis reduces metastasis formation in our metastatic UM zebrafish model whereby GPX4 expression levels seem to be indicative of ferroptosis susceptibility in vivo, and therefore could be predictive of patient response. [46, 47].

Taken together, we provide a simple and fast in vivo model for drug screening against human UM and provide evidence that ferroptosis induction is a promising strategy to treat UM in BAP1^-^/monosomy 3 patients.

## Materials and methods

### Adherent culture of UM cells

All UM cell lines (MP46, MM28, Xmm66 [30], Omm1 [31], Mel285, Omm2.3 [48] as well as here generated) were cultured in Dulbecco’s modified eagle’s medium (DMEM) containing 10% fetal bovine serum (FBS, Gibco, Thermo Fisher Scientific, Waltham, MA USA), supplemented with Glutamax (Gibco). Cell lines were cultured (<20) passages and intermittently checked for the presence of mycoplasma.

### Establishment of spheroid cultures

Metastatic UM patient tissues that were frozen in neuronal stem cell medium (NSC medium, Stemcell technologies, Köln, Germany) containing 10% DMSO were thawed by brief incubation at 37 °C and were transferred to NSC medium without growth factors. Prior to the generation of UM spheroids, the medium was exchanged for 10 ml NSC medium containing 5 mg/mL Primocin (Invivogen, Toulouse, France). Subsequently the tissue was minced using a sterile scalpel blade and was transferred into a 50 ml centrifuge tube with 10 ml of NCS medium supplemented with 0.01 mg/ml Liberase TL (Roche, Woerden, the Netherlands). The tissue was dissociated by incubation at 37 °C for 3–5 h while shaking at 250 rpm and intermittent vortexing. The cell suspension was then passed through a sterile 30 µm cell strainer to remove all cell and extracellular matrix aggregates. Cells were pelleted and suspended in complete NSC medium (supplemented with 1x B27 (Gibco), 1x N2 (Gibco), 20 ng/ml bFGF (Peprotech, Hamburg, Germany), 20 ng/ml EGF (Peprotech), 5 U/ml heparin, 1x primocin (Invivogen), 5% FBS, 200 mM Glutamax). The cells of 0.25 cm^3^ original tumor volume were plated in ~8 wells of a 24-well ultra-low attachment plate (Corning, Wiesbaden, Germany). Culturing at 37 °C and 5% CO_2_ resulted in spheroid formation within 24 h. Sphere cultures were kept in complete NSC medium in ultra-low adhesion well plates, and were subjected to medium changes every 3 days.

### Dissociation and staining of UM spheroid culture-derived cells prior to engraftment

Spheroids were collected (approximately 6–8 wells of a 24 well plate) through centrifugation (200 × g, 5 min, room temperature) and resuspended in 3 ml TrypLE (Gibco). After a 10 min incubation at 37 °C, combined with intermittent agitation with a 1000 µl pipette, aggregates were broken up. Then, TrypLE was inactivated by addition of 7 ml complete NSC medium. Following centrifugation (200 × g, 5 min, room temperature), the red fluorescent lipid dye CM-DiL (Sigma) was used to stain the cells to visualize cancer cell proliferation and metastatic initiation as reported previously [49].

### Lentiviral transduction

Both adherent cell culture and spheroid cultures were lentivirally transduced as previously described [50]. In brief, the adherent cells were cultured in the presence of lentiviral particles containing ΔLTR flanked CMV:tdTomato-blasticidin (Addgene#106173) and 8 µg/ml polybrene (Sigma, Zwijndrecht, the Netherlands). Subsequently the medium was exchanged for standard culture medium. Transduced adherent UM lines were selected with 2 µg/ml blasticidin (Gibco). The procedure was the same for transduction of cultured spheroids, except for the generation of a single cell suspension, by incubation with TrypLE, for 5 min at 37 °C followed by mechanical dissociation by repeated pipetting, prior to the addition of the viral particles.

### Zebrafish maintenance

All animal experiments were performed according to Dutch Animal Protection Laws approved by the local governmental animal protection committee. Embryonic and adult zebrafish were raised and maintained in custom built glass aquaculture systems (main and stand-alone, Zebcare, Nederweert, The Netherlands) according to the guidelines specified on ZFIN.org. Zebrafish were fed three times a day, depending on age, with rotifers and granular food. Health monitoring was performed at least once per year. Zebrafish larvae were collected and maintained in zebrafish medium (de-ionized water containing “Instant Ocean” Sea Salts Instant Ocean, Blacksburg, VA, USA, at a final concentration of 60 µg/ml).

### Injection of cancer cells into zebrafish

Either *Tg(fli:GFPx casper)* (ZFIN ID: ZDB-TGCONSTRCT-070117-94 crossed with ZFIN ID: ZDB-GENO-080326-11) [45, 51, 52] fish were bred prior to the start of an experiment, and Petri dishes containing larvae were cleaned every day, removing dead and malformed larvae, after harvesting up to 2 days post fertilization. Approximately 300 to 400 cells were injected into the Duct of Cuvier (doC, the embryonic common cardinal vein) of 2 dpf zebrafish larvae as described [49].

### Imaging of zebrafish xenografts

For each drug treatment experiment 20 injected zebrafish larvae were randomly selected and imaged using a MZ16FA fluorescence microscope equipped with a DFC420C camera (Leica, Wetzlar, Germany) as described [49]. Representative engrafted phenotypes were subjected to confocal imaging; to this end zebrafish larvae were anaesthetized with 0.002% tricaine (MS222, Sigma) in zebrafish medium. After embedding the larvae in 1% low melting temperature agarose/zebrafish medium, images were recorded for both green (GFP) and red (tdTomato/CMDiI) channels. Whole larva stitches were generated at 10x magnification using a Leica sp8 confocal microscope (Leica, Wetzlar, Germany). Consecutive stitch sequences were processed into a single image using Fiji 30 using a previously described plugin [53]. Metastatic capacity or tumor burden at the end of the experiment was determined as the normalized fluorescence intensity of the engrafted cells (either normalized to 1 dpi for time course experiments or to vehicle treated control for treatment experiments).

### Small molecule inhibitors/drugs

All drugs were acquired from Cayman chemical (Ann Arbor, Michigan, USA) and were dissolved in dimethyl sulfoxide (DMSO) unless otherwise stated. Everolimus: mTORC1 inhibitor (RAD001, Item No. 11597); sotrastaurin: PKC inhibitor (AEB071, Item No. 16726); navitoclax: BCL-2/BCL-xl inhibitor (ABT263, Item No. 11500); quisinostat: HDAC inhibitor (Item No. 14088); flavopiridol: CDK inhibitor (Item No. 26024). RSL3 ((1 S,3 R)-RSL3, Item No. 19288), and erastin (Item No. 17754), both ferroptosis inducers by inhibiting system Xc- and VDAC1/2 or GPX4, respectively.

### Establishment of maximum tolerated drug dose in zebrafish

The maximum tolerated dose (MTD) of drugs in zebrafish was determined as previously described [45]. In brief, 10 µM to 156 nM of a small molecule inhibitor was added to zebrafish larvae at 3 dpf (corresponding to 1 dpi in injected larvae). The compound was refreshed every other day and survival was scored at 8 dpf (corresponding to 6 dpi in engrafted individuals). The highest concentration with at least 80% survival was chosen as MTD. Note, for combinatorial treatments, we titrated from MTD A (determined prior) combined with 10 µM to 156 nM compound B to attain a suitable treatment concentration where >80% of all treated larvae survived up to 8 dpf for a 6-day treatment (starting at 2 dpf).

### Drug treatment of UM engrafted zebrafish

At 16 h post injection, larvae were screened for proper engraftment and absence of abnormal phenotypes (edema, necrosis) and clear presence of disseminated cells throughout the larvae. Subsequently, larvae were randomly subdivided into a 24-well plate, with 6 individuals per well and 6 wells per condition. The compounds were dissolved in DMSO and diluted in zebrafish medium and added. The volume of vehicle (DMSO) control used was the same as the highest volume of drug added.

### Clinical data analysis

The Leiden University Medical Centre (LUMC) cohort includes clinical, histopathological, and genetic information on 64 UM cases, enucleated between 1999 and 2008. Clinical information was collected from the Integral Cancer Center West patient records and updated in 2019. For each sample, part of the tumor was snap frozen with 2-methyl butane and used for mRNA and DNA isolation, while the remainder was embedded in paraffin after 48 hours of fixation in 4% neutral-buffered formalin and sent for histological analysis. Chromosome status was determined with the Affymetrix 250K_NSP-chip and Affymetrix Cytoscan HD chip (Affymetrix, Santa Clara, California, United States of America). RNA was isolated with the RNeasy mini kit (Qiagen, Venlo, The Netherlands) and mRNA expression was determined with the HT-12 v4 chip (Illumina, San Diego, California, United States of America). Statistical analyses of the LUMC cohort were carried out in SPSS, version 25 (IBM Corp). For survival analysis, Kaplan-Meier and log-rank test were performed with death due to metastases as endpoint. Cases that died of another or unknown cause were censored. The two subpopulations that were compared in each analysis were determined by splitting the total cohort along the median value of mRNA expression for each analyzed gene.

### Immunohistochemical analysis of engrafted zebrafish larvae

Engrafted zebrafish were euthanized with tricaine overdosing (300 mg/L in zebrafish medium, for 10 minutes) and fixed for 16 h in ice-cold 4% paraformaldehyde in PBS. After fixation, larvae were washed with PBS containing 0.05% tween 20 (v/v) and 200 mM glycine. Zebrafish were aligned in *x,y* and *z* axes in agarose prior to embedding in paraffin. After paraffin-embedding, 4 µm thin sections were cut and placed onto X-tra adhesive slides (Leica Biosystems, Milton Keynes, UK). Immunohistochemical staining was performed using a Bond RXm Automated Stainer with high pH antigen retrieval and the Bond polymer-refine detection systems in either red or brown chromogen, according to the manufacturers’ recommendations (Leica Biosystems). Primary antibodies included mouse anti-melanA (Dako, Agilent, Cheshire UK) and mouse anti-BAP1 (Santa Cruz Biotechnology, USA), both at a concentration of 1 µg/ml. Slides were counterstained with hematoxylin and mounted with a resin-based mounting agent. Human UM tissue was used as a positive control for each of the primary antibodies. Mouse IgG1 isotype control at a concentration of 1 µg/ml was also included in each assay.

### qPCR analysis

Whole RNA was isolated from 1 × 10^6^ cells using the Qiagen RNeasy kit (Qiagen) and cDNA was synthesized using the iSCRIPT cDNA kit (Biorad, Hercules, USA), according to the manufacturer’s description. Expression levels were determined with an iQ5 qPCR apparatus (Biorad) utilizing IQ green super mix (Biorad) with 35 cycles. A description of all used primers can be found in Supplementary Table 2. Glyceraldehyde-3-phosphate dehydrogenase (GAPDH) and Calpain Small Subunit 1 (CAPNS1) levels were used as an internal reference for each experimental primer set.

### Protein lysates and western blotting

To determine protein expression in UM cell cultures, cells were seeded into 6-well plates. After two days, when cells were ~70–80% confluent, they were rinsed twice with ice-cold PBS on ice and subsequently lysed in Giordano buffer (50 mM Tris-HCl at pH 7.4, 250 mM NaCl, 0.1% Triton X-100, 5 mM EDTA; supplemented with protease- and phosphatase inhibitors) for 10 min on ice. After scraping and transferring lysates to tubes, lysates were centrifuged for 15 min at 3000 × *g*. Supernatant was transferred to a clean tube. Lysates of primary UM samples were made in Giordano buffer. After crushing nitrogen-frozen pieces of tumor to powder, the samples were processed as described for cell cultures. Protein concentrations were determined with Bradford reagent (Bio-Rad). Equal amounts of proteins were separated on SDS-polyacrylamide gels, and proteins were transferred onto PVDF membranes (Millipore). After blocking in 10% non-fat dry milk in TBST (10 mM Tris-HCl, pH 8.0, 150 mM NaCl, 0.2% Tween-20), the blots were incubated overnight, at 4 °C with antibodies diluted in TBST/5% bovine serum albumin (BSA). After washing with TBST and incubation with secondary antibodies coupled to HRP diluted in TBST for 30 min, blots were washed thoroughly and imaged using a Chemidoc (Bio-Rad). The following antibodies were used: anti-GPX4 (clone B12, mouse anti-human monoclonal, dilution 1:200), anti-BAP1 (clone C4, monoclonal mouse anti-human, dilution 1:200) (both Santa Cruz Biotechnology), anti-SLC7A11 (clone D2M7A, monoclonal rabbit anti-human, dilution 1:1000), anti-ERK1/2 (clone L34F12, monoclonal mouse anti-human, dilution 1:2000) both from Cell Signaling Technology, anti-Vinculin (clone V9131, monoclonal mouse anti-human, dilution 1:1000) both from Sigma-Aldrich. Original blots are shown in Supplementary Fig. 3.

### In vitro growth assay

To investigate the effect(s) of inducers (Erastin and RSL3) and inhibitors of ferroptosis (Ferrostatin-1 and Liproxstatin, both 10 µM) on cell survival in vitro, cell lines were seeded in triplicate or quadruplicate in 96-well plates. The next day, cells were treated with the different compounds. Survival was determined after 5 days of incubation using the Cell Titer-Blue assay (Promega). All cell lines were treated with 4 and 8 µM Erastin and 3 and 6 µM RSL3, with the exception of Mel285 which was treated with 0.05 and 0.2 µM Erastin or RSL3. Results are shown Supplementary Fig. 4.

### Statistical analysis

Prior to normalization and combination of all biological replicates for statistical analysis, outliers were removed from all data sets using Graphpad Prism 8.0 (Q5). Then, data were normalized to either control (drug treatment) or to day one (in growth kinetic experiments). Statistical significance was tested with a one-way ANOVA. Data are presented as mean ± SD, data was tested for normalcy prior to statistical assessment and variance was similar between the groups that were statistically compared, p-values ≤ 0.05 are considered to be statistically significant (*:*p* < 0.05, **:*p* < 0.01, ***:*p* < 0.001).

## Supplementary information

Supplementary Fig. 1 Determining the maximum tolerated dosage of applied drugs in the metastatic uveal melanoma zebrafish model.

Supplementary Fig. 2 Ferroptosis-related genes are strongly associated with a bad prognosis in uveal melanoma.

Supplementary Fig. 3

Supplementary Fig. 4 Induction of ferroptosis significantly reduces cell survival in vitro.

Supplementary Fig. 5 Representative images of ferroptosis inducer treated zebrafish xenograft models.

Original Data File

Supplementary Table 1

Supplementary Table 2

in vivo ferroptosis induction data

## Data Availability

Data are available from the corresponding author upon reasonable request.
