# Supplementary material for: Patient-derived zebrafish xenografts of uveal melanoma reveal ferroptosis as a drug target
Source: Cell Death Discov. 2023 Jun 16;9:183. doi: 10.1038/s41420-023-01446-6 (PMC10272172; doi:10.1038/s41420-023-01446-6)
Supplement: Supplementary file 1 — Supplementary Fig. 1 Determining the maximum tolerated dosage of applied drugs in the metastatic uveal melanoma zebrafish model. [file 41420_2023_1446_MOESM1_ESM.docx]

**
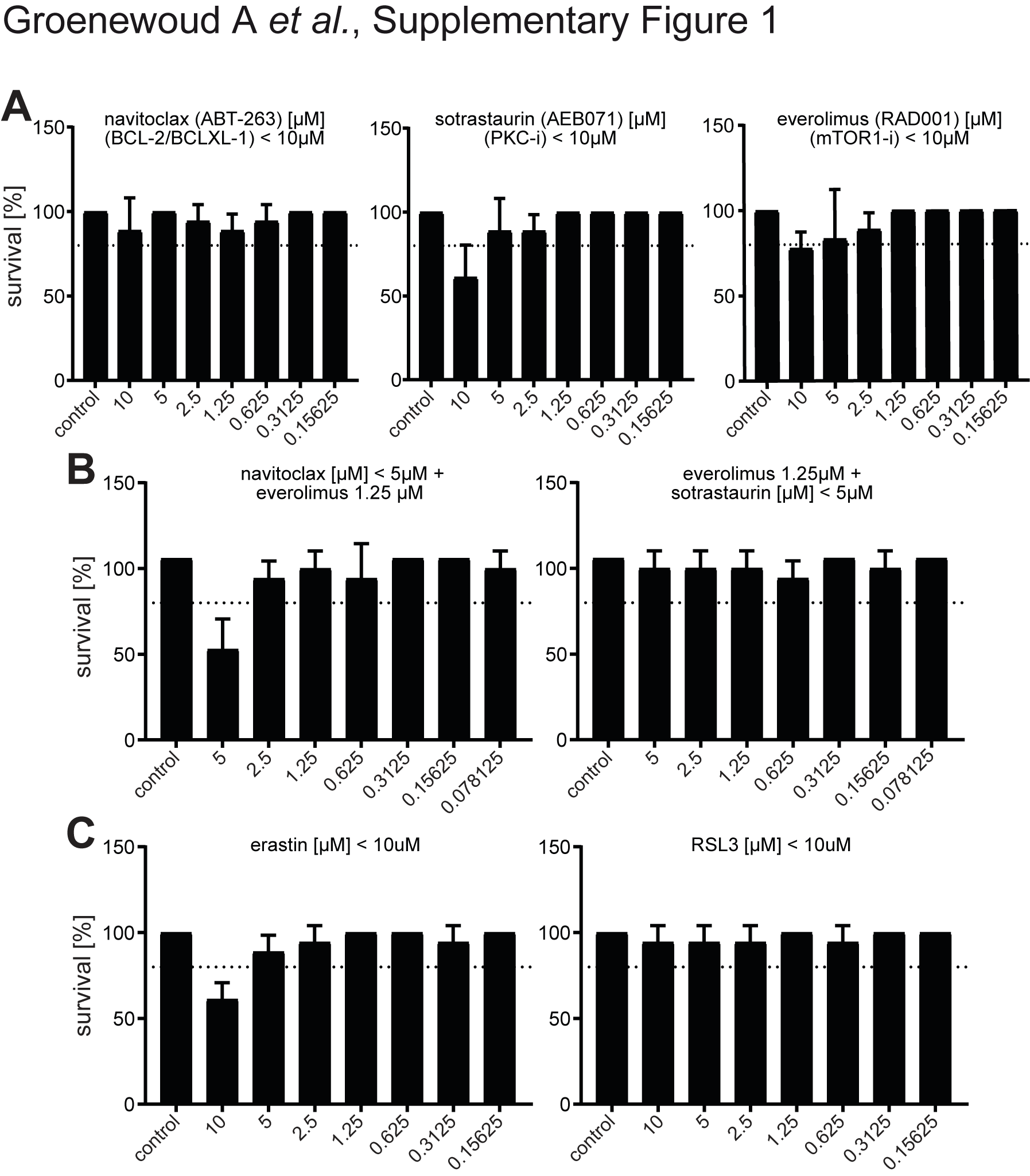
**

**Supplementary Fig. 1 Determining the maximum tolerated dosage of applied drugs in the metastatic uveal melanoma zebrafish model. A**-**C** Assessment of the survival rate of 48 hpf wildtype or *Tg(fli:GFP x casper)* zebrafish larvae treated for 5 days (treatments were refreshed every other day) with single putative anti-uveal melanoma therapeutics (**A**) or combinations thereof (**B**) or with ferroptosis inducing compounds (**C**) at the indicated concentrations. The maximum tolerated dosage was defined as the dose at which at least 80% of the treated zebrafish larvae survived for the duration of the treatment. Data are mean ± SD. n = 18.
