# Supplementary material for: Patient-derived zebrafish xenografts of uveal melanoma reveal ferroptosis as a drug target
Source: Cell Death Discov. 2023 Jun 16;9:183. doi: 10.1038/s41420-023-01446-6 (PMC10272172; doi:10.1038/s41420-023-01446-6)
Supplement: Supplementary file 2 — Supplementary Fig. 2 Ferroptosis-related genes are strongly associated with a bad prognosis in uveal melanoma. [file 41420_2023_1446_MOESM2_ESM.docx]

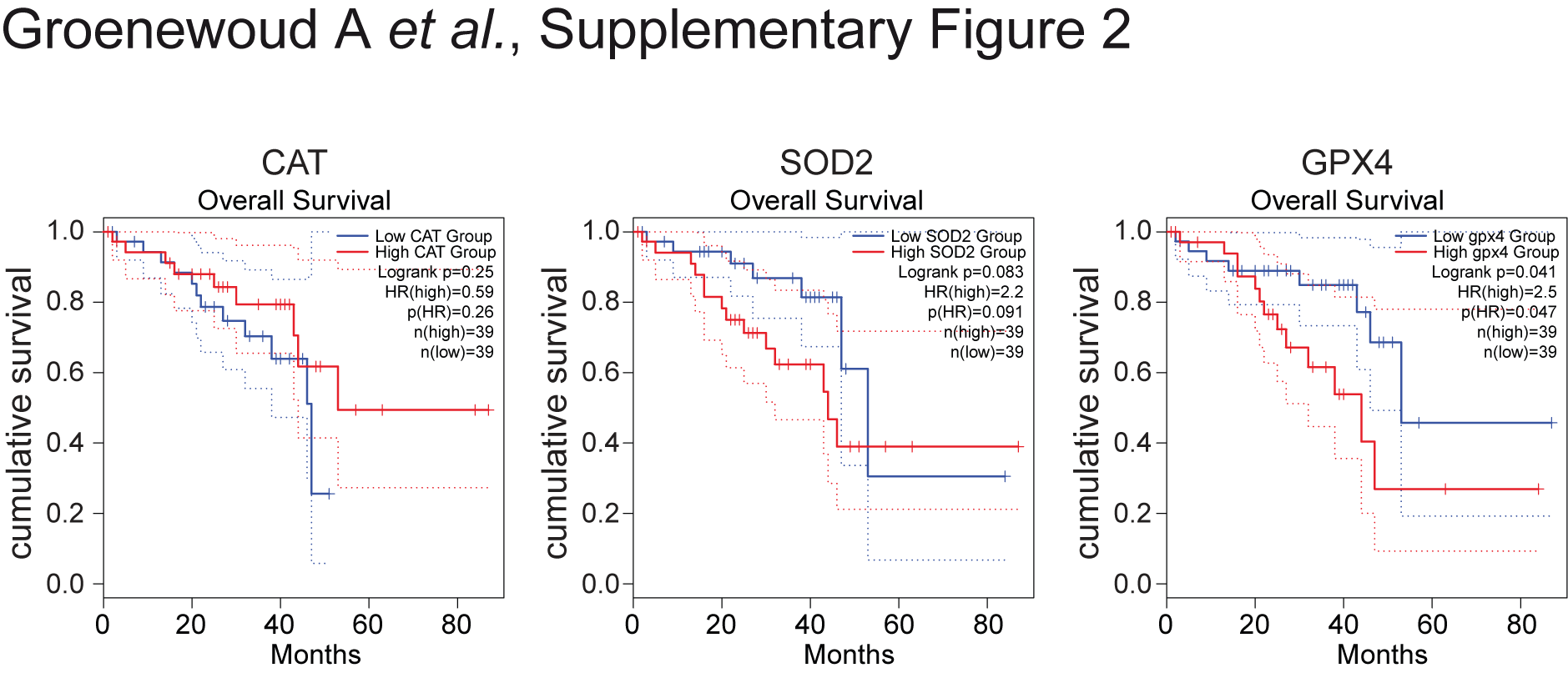


**Supplementary Fig. 2 Ferroptosis-related genes are strongly associated with a bad prognosis in uveal melanoma.** Analysis of the cancer genome atlas (TCGA) revealed that of the three major ROS detoxifying enzymes catalase (CAT), superoxide dismutase2 (SOD2) and glutathione peroxide 4 (GPX4), GPX4 is the only one that correlates significantly with a bad prognosis.
