# Supplementary figures and images for: Patient-derived zebrafish xenografts of uveal melanoma reveal ferroptosis as a drug target
Source: Cell Death Discov. 2023 Jun 16;9:183. doi: 10.1038/s41420-023-01446-6 (PMC10272172; doi:10.1038/s41420-023-01446-6)

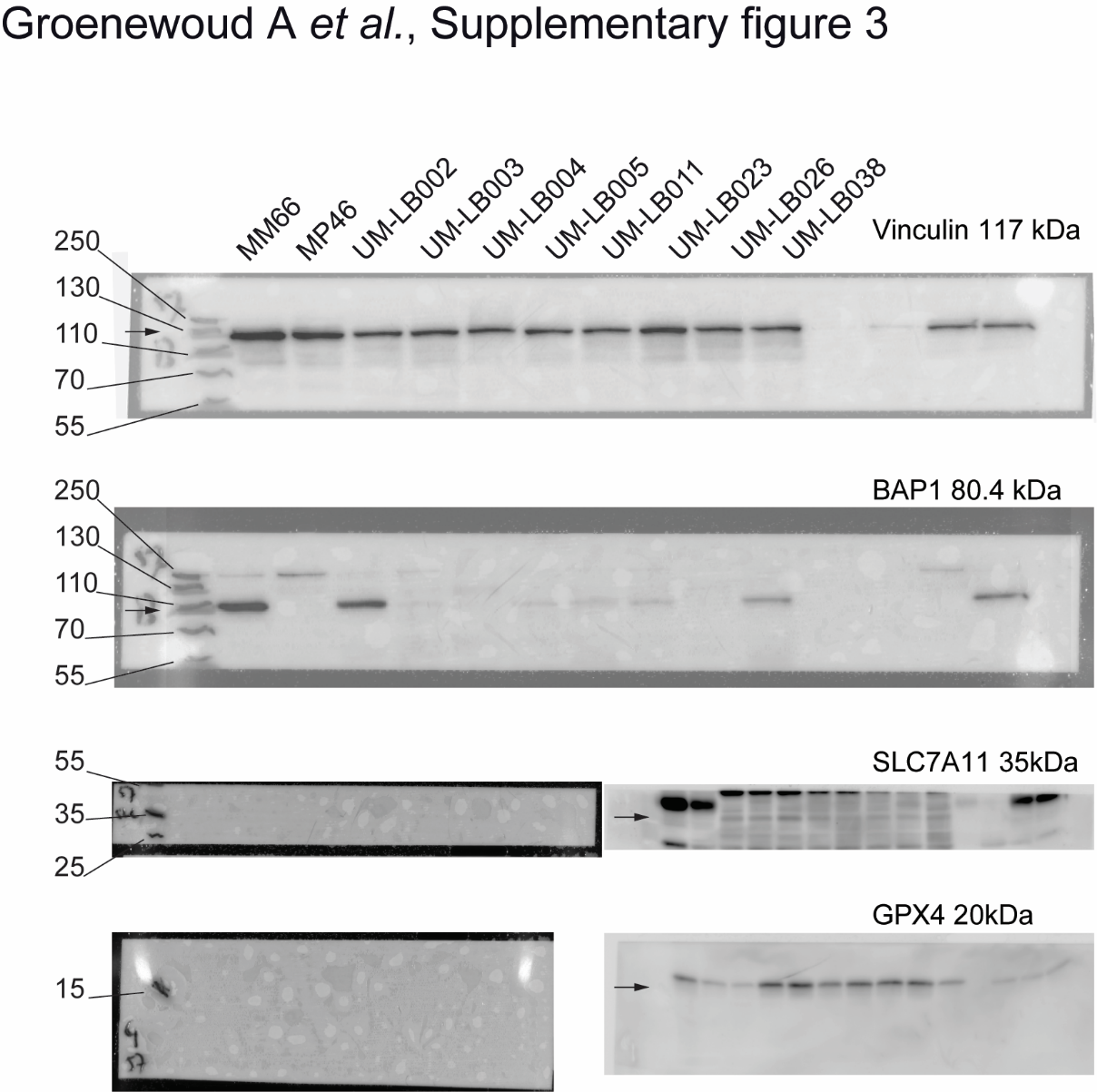


**Supplementary Fig. 3** Full western blots used for Fig. 5A.

Supplement: Supplementary file 3 — Supplementary Fig. 3 [file 41420_2023_1446_MOESM3_ESM.docx]

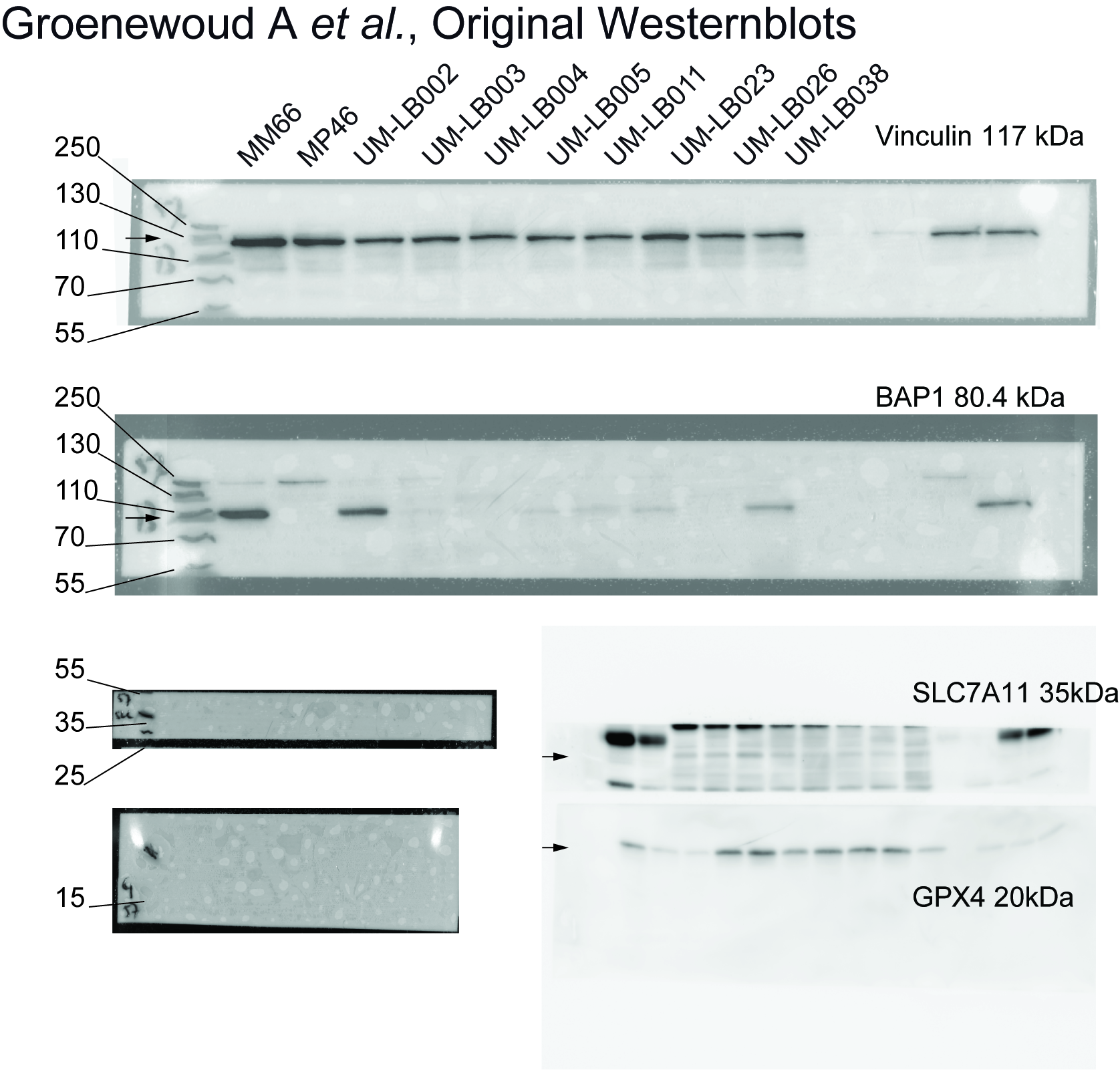

Supplement: Supplementary file 6 — Original Data File [file 41420_2023_1446_MOESM6_ESM.tif]
