# Supplementary material for: Patient-derived zebrafish xenografts of uveal melanoma reveal ferroptosis as a drug target
Source: Cell Death Discov. 2023 Jun 16;9:183. doi: 10.1038/s41420-023-01446-6 (PMC10272172; doi:10.1038/s41420-023-01446-6)
Supplement: Supplementary file 4 — Supplementary Fig. 4 Induction of ferroptosis significantly reduces cell survival in vitro. [file 41420_2023_1446_MOESM4_ESM.docx]

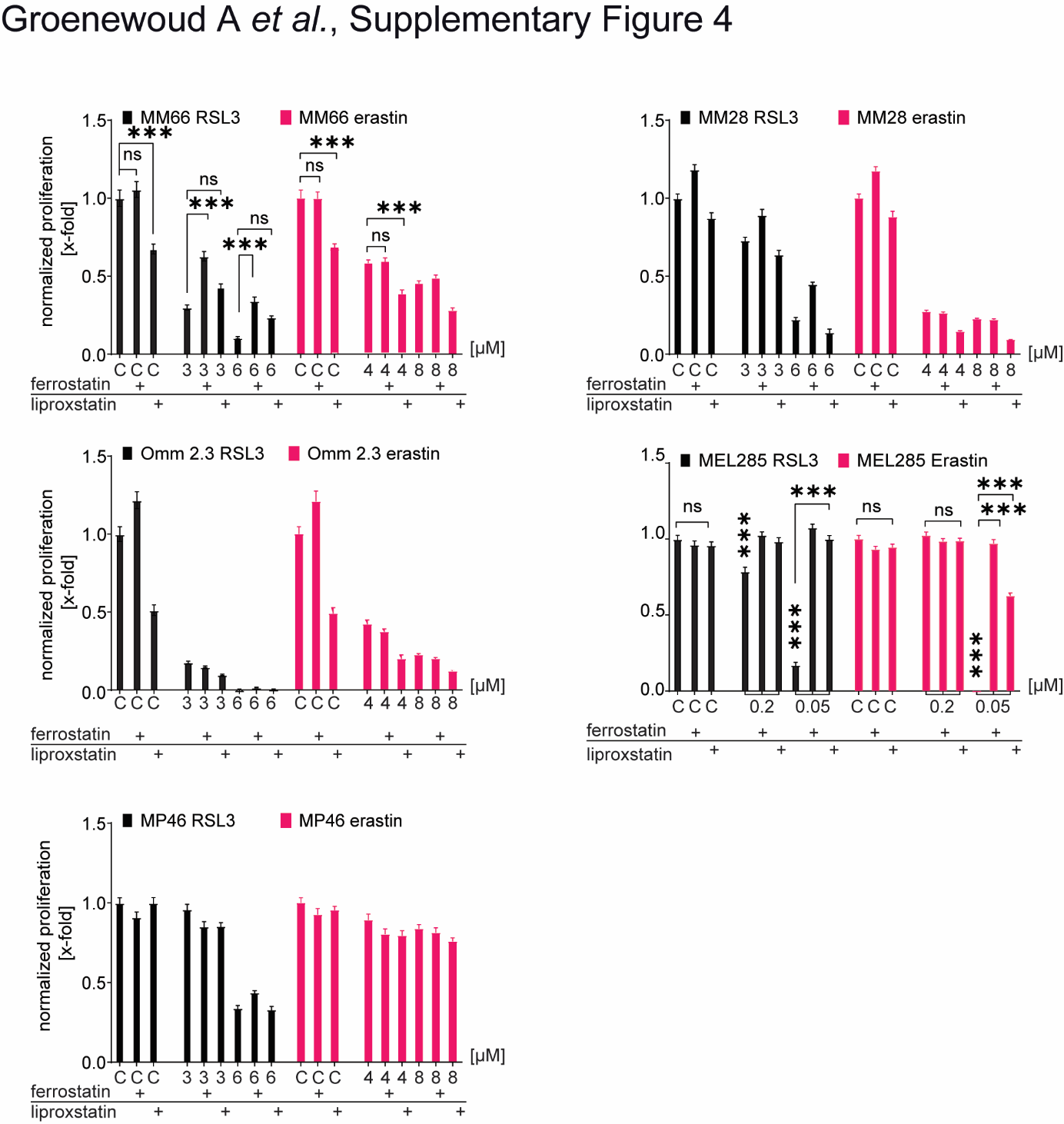


**Supplementary Fig. 4 Induction of ferroptosis significantly reduces cell survival in vitro.** A) *In vitro* treatment of primary (MP46) and metastatic (Omm1, mm28 and Xmm66) uveal melanoma. All cell lines were treated with 8 and 4 µM Erastin and 6 and 3 µM RSL3, with the exception of MEL285 which was treated with 0.2 and 0.05 µM Erastin or RSL3 and subsequent rescue was attempted with ferroptosis inhibitors ferrostatin and liproxstatin (both at 10 µM).
