# Supplementary material for: Patient-derived zebrafish xenografts of uveal melanoma reveal ferroptosis as a drug target
Source: Cell Death Discov. 2023 Jun 16;9:183. doi: 10.1038/s41420-023-01446-6 (PMC10272172; doi:10.1038/s41420-023-01446-6)
Supplement: Supplementary file 5 — Supplementary Fig. 5 Representative images of ferroptosis inducer treated zebrafish xenograft models. [file 41420_2023_1446_MOESM5_ESM.docx]

**
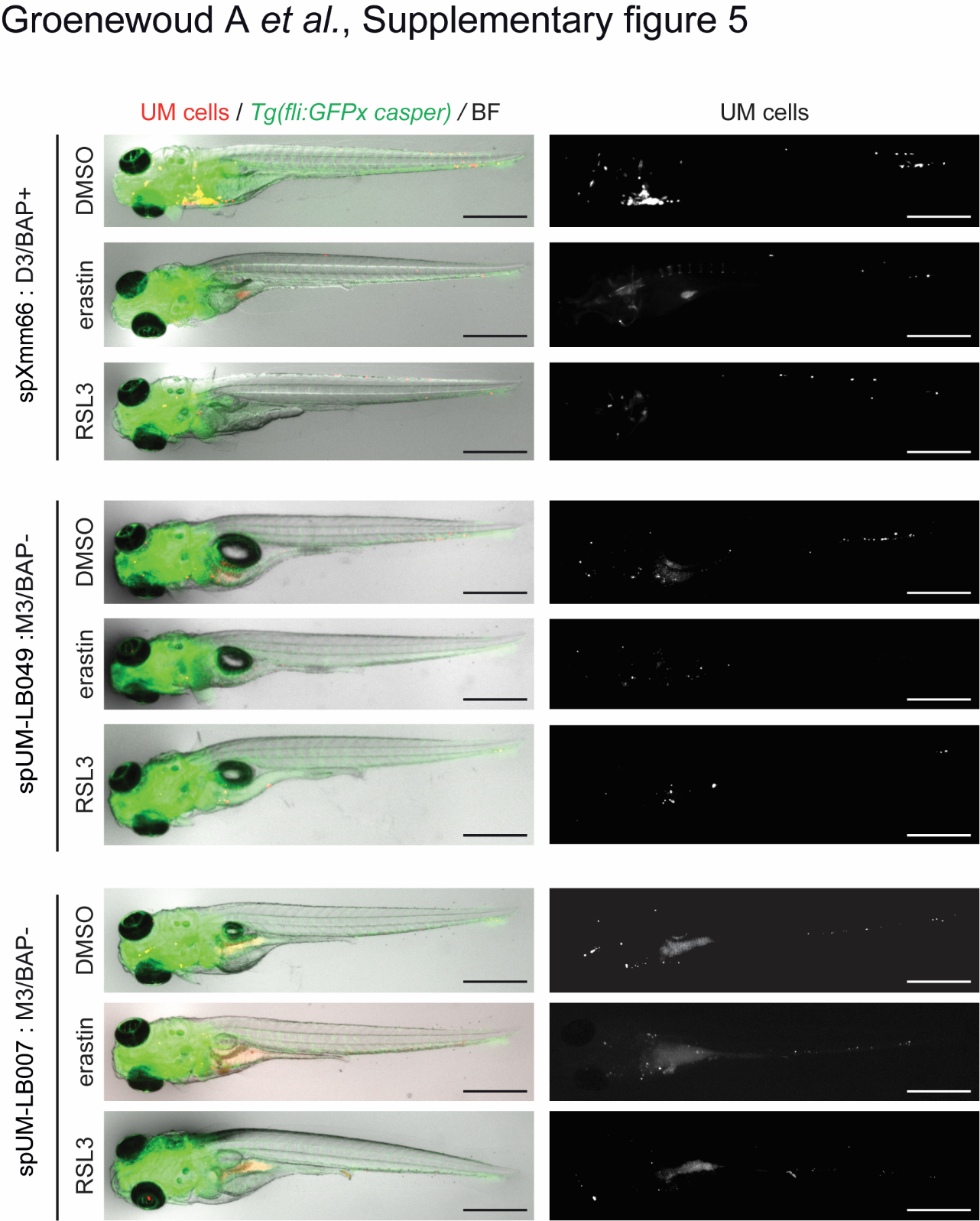
**

**
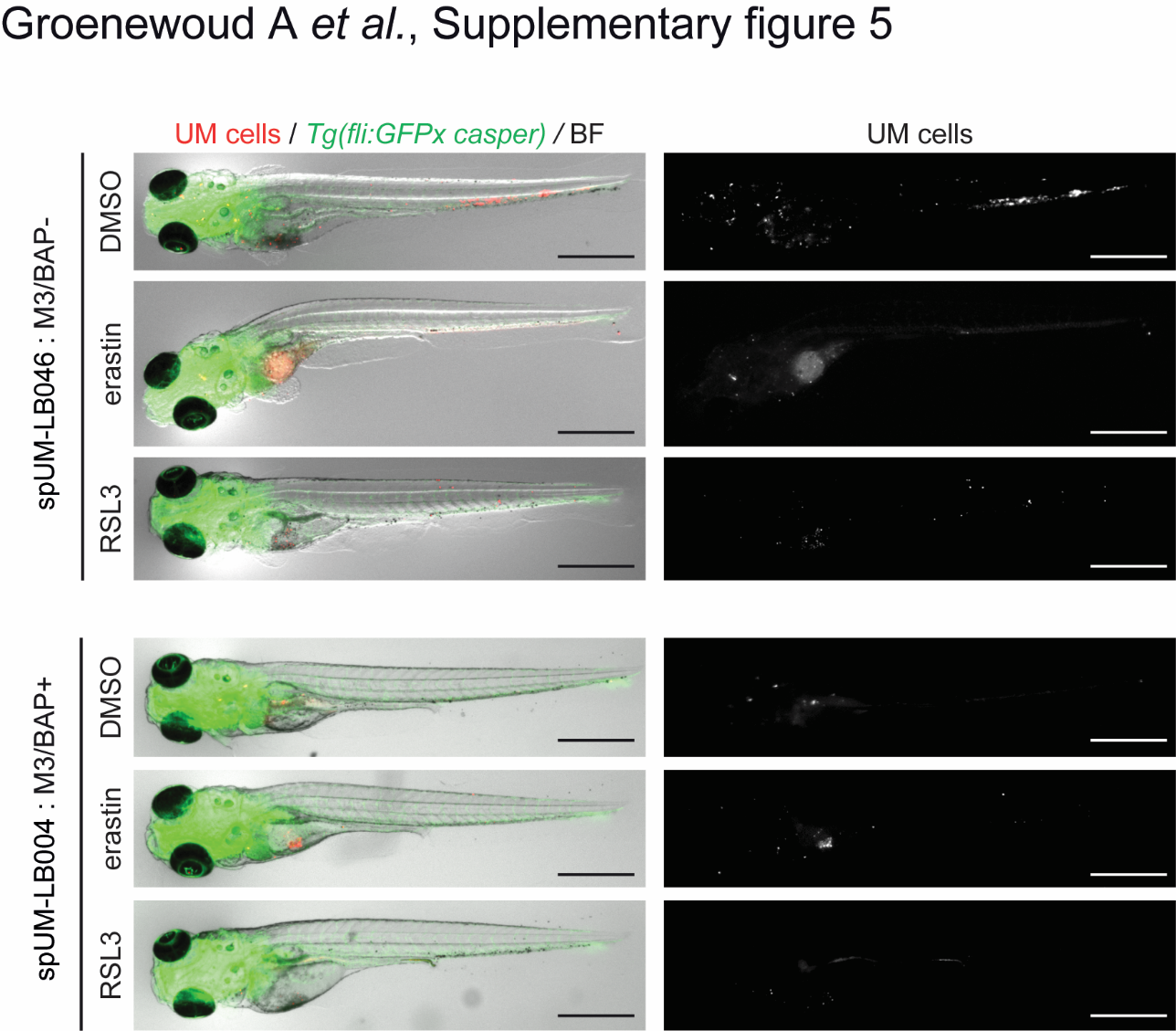
**

**Supplementary Fig. 5 Representative images of ferroptosis inducer treated zebrafish xenograft models.** For each drug treatment all surviving (± 20) zebrafish larvae were imaged using a MZ16FA fluorescence microscope equipped with a DFC420C camera. Out of these approximately 20 larvae, one representative larva was selected per treatment group and shown (scale bar = 500 um).
