# Supplementary material for: Patient-derived zebrafish xenografts of uveal melanoma reveal ferroptosis as a drug target
Source: Cell Death Discov. 2023 Jun 16;9:183. doi: 10.1038/s41420-023-01446-6 (PMC10272172; doi:10.1038/s41420-023-01446-6)
Supplement: Supplementary file 7 — Supplementary Table 1 [file 41420_2023_1446_MOESM7_ESM.docx]

Groenewoud et al., Supplementary Table 1

|  | **Name** | **Recovered from**  **freezing** | **Sphere culture established** | **Lentiviral trans-duction** | **Zebrafish Xenograft** | **Vali-dation** | **Growth speed** | **Max. passage** | **Reference(s)** |
| --- | --- | --- | --- | --- | --- | --- | --- | --- | --- |
| metastatic | MM26 | ✓ | ✓ | - | ✓ | S, IHC, C | -/± | 4 | Nemati, Laurent |
|  | MM28 | ± | ± | - | IM | N/A | - | 4 | Nemati, Amirouchene |
|  | MM33 | ✓ | ✓ | - | IM | S | -/± | 4 | Nemati, Laurent, Carita |
|  | MM52 | ✓ | ✓ | - | IM | N/A | -- | 4 | Nemati, Laurent, Carita |
|  | MM66 | ✓ | ✓ | ✓ | ✓ | S, IHC, C, D | + | 20+ | Nemati, Laurent, Amirouchene |
|  | MM252 | N/A | ✓ | - | IM | S | N/A | N/A | N/A |
|  | MM257 | N/A | ✓ | - | IM | S | N/A | N/A | N/A |
|  | MM267 | ✓ | ✓ | ✓ | IM | S | N/A | N/A | N/A |
|  | MM278 | N/A | ✓ | - | IM | S | N/A | N/A | N/A |
|  | MM293 | ✓ | ✓ | ✓ | IM | S | N/A | N/A | N/A |
|  | MM299 | N/A | ✓ | - | IM | S | N/A | N/A | N/A |
|  | MM300 | N/A | ✓ | ± | IM | S | N/A | N/A | N/A |
|  | MM309 | ✓ | ✓ | ✓ | IM | S | N/A | N/A | N/A |
|  | MM325 | ✓ | ✓ | ✓ | IM | S | N/A | N/A | N/A |
| primary | UM 17-045 | ✓ | ✓ | N/A | - | S | N/A | N/A | N/A |
|  | UM 17-046 | ✓ | ✓ | ✓ | ✓ | S, D | N/A | N/A | N/A |
|  | UM 17-047 | ✓ | ✓ | N/A | ✓ | S, C | N/A | N/A | N/A |
|  | UM 17-048 | ✓ | ✓ | N/A | ✓ | S, C, D | N/A | N/A | N/A |
|  | UM 17-049 | ✓ | ✓ | N/A | - | S | N/A | N/A | N/A |
|  | UM 18-004 | ✓ | ✓ | N/A | ✓ | S, D | N/A | N/A | N/A |
|  | UM 18-005 | ✓ | ✓ | N/A | - | S | N/A | N/A | N/A |
|  | UM 18-007 | ✓ | ✓ | N/A | ✓ | S, D | N/A | N/A | N/A |
|  | UM 18-008 | ✓ | ✓ | N/A | - | S | N/A | N/A | N/A |
|  | UM 18-010 | ✓ | ✓ | N/A | - | S | N/A | N/A | N/A |

S = Sphere culture; IHC = Immunohistohemistry; C = confocal imaging;

D = Drug screen; IM = insufficient material
