# Supplementary material for: Patient-derived zebrafish xenografts of uveal melanoma reveal ferroptosis as a drug target
Source: Cell Death Discov. 2023 Jun 16;9:183. doi: 10.1038/s41420-023-01446-6 (PMC10272172; doi:10.1038/s41420-023-01446-6)
Supplement: Supplementary file 8 — Supplementary Table 2 [file 41420_2023_1446_MOESM8_ESM.docx]

Groenewoud et al., Supplementary Table 2

| **Gene** | **Forward primer** | **Reverse primer** |
| --- | --- | --- |
| GAPDH | 5‘-TCAAGGCTGAGAACGGGAAG-3‘ | 5‘-CGCCCCACTTGATTTTGGAG-3‘ |
| CAPNS1 | 5‘-ATGGTTTTGGCATTGACACATG-3‘ | 5‘-GCTTGCCTGTGGTGTCGC-3‘ |
| MITF | 5'-AACAGAGAGTGCCCGTGAGT-3' | 5'-GACATGGCAAGCTCAGGACT-3' |
| TYR | 5'-TACGGCGTAATCCTGGAAAC-3' | 5'-ATTGTGCATGCTGCTTTGAG-3' |
| DCT | 5'-GGGAGGAACGAGTGTGATGT-3' | 5'-TGGCAATTTCATGCTGTTTC-3' |
| TYRP1 | 5'-CTGGAATTTTGCAACGGGGA-3' | 5'-CCATCCTCGGTGCTGTTACA-3' |
| SOX10 | 5'-CTTCATGGTGTGGGCTCAG-3' | 5'-TGTAGTCCGGGTGGTCTTTC-3' |
| GPX4 | 5'-TGGACAAGTACCGGGGCTTC-3' | 5'-CGAACTGGTTACACGGGAAG-3' |
| SCL7A11 | 5'-TGCTGTGATATCCCTGGCAT-3' | 5'-AGCTGCATAACTCCAGGGAC-3' |
